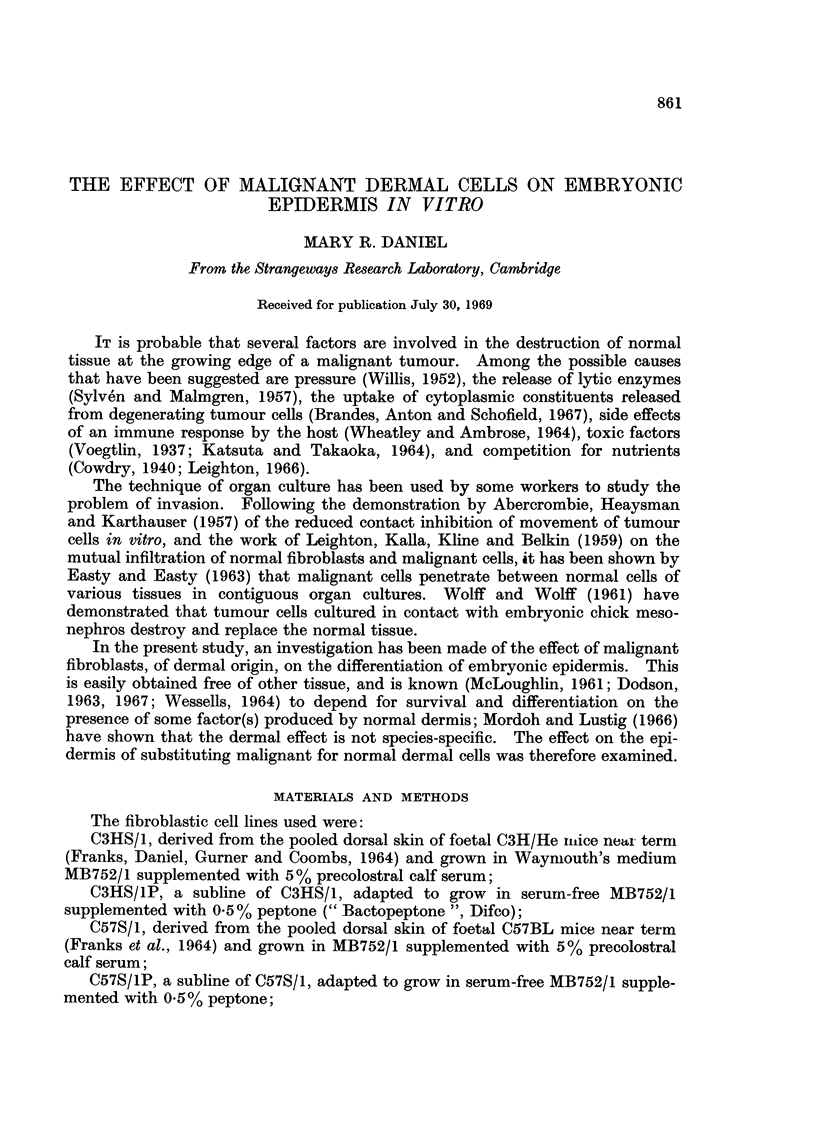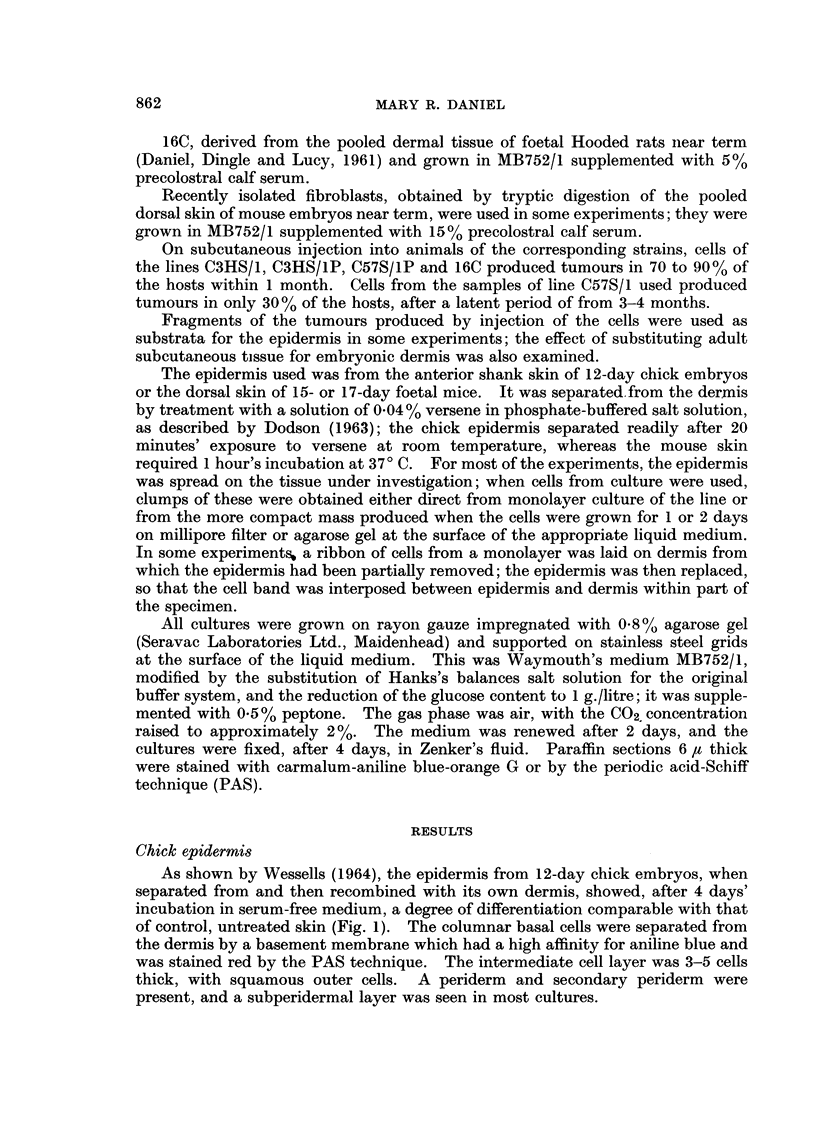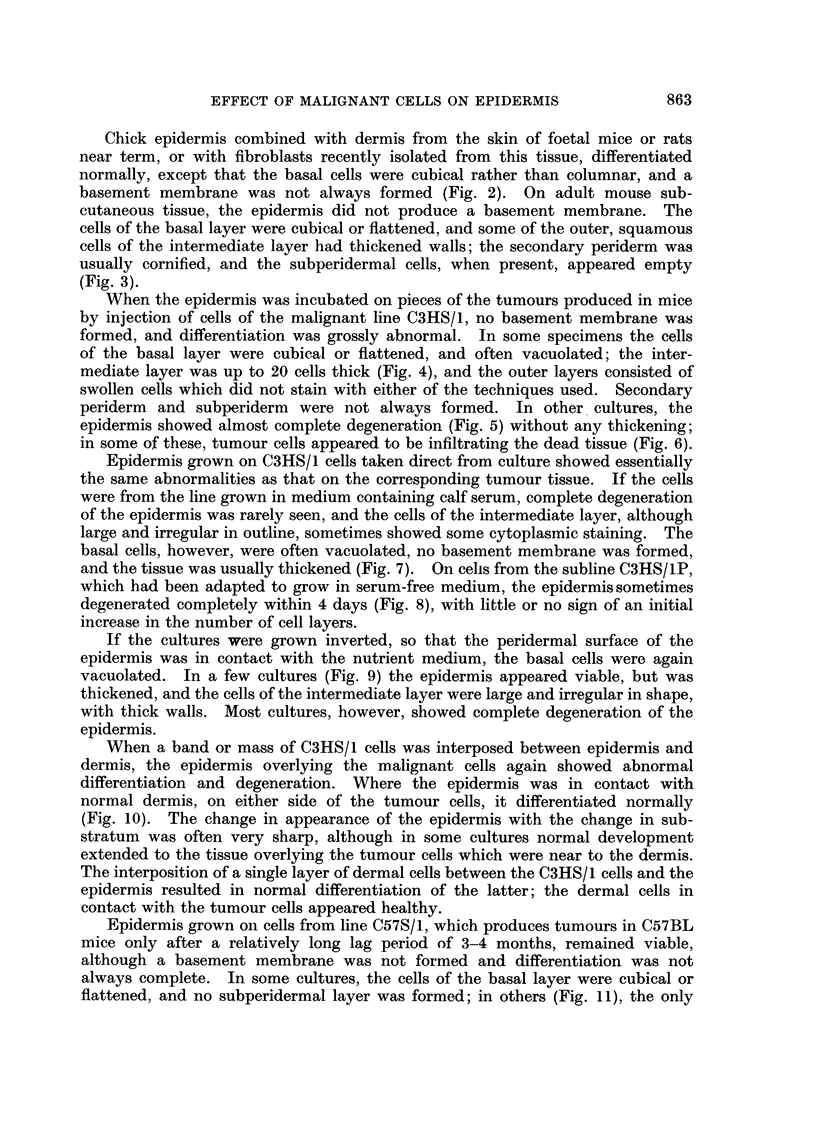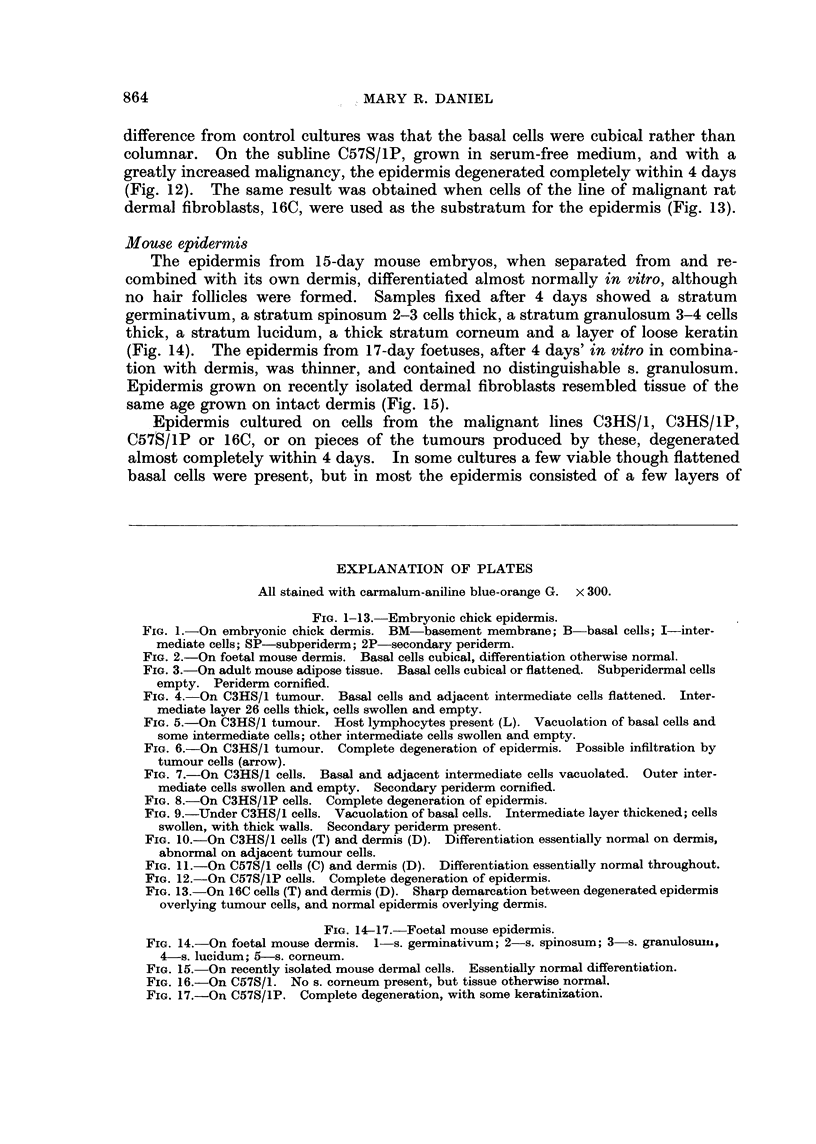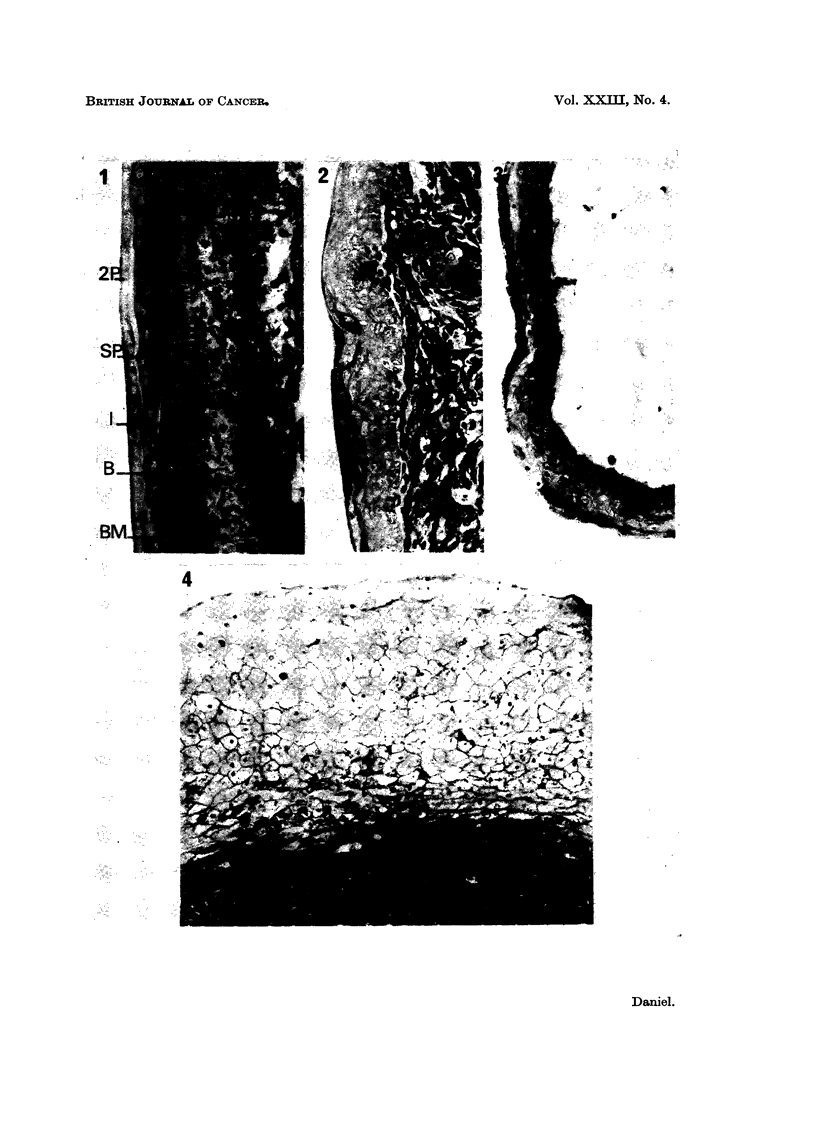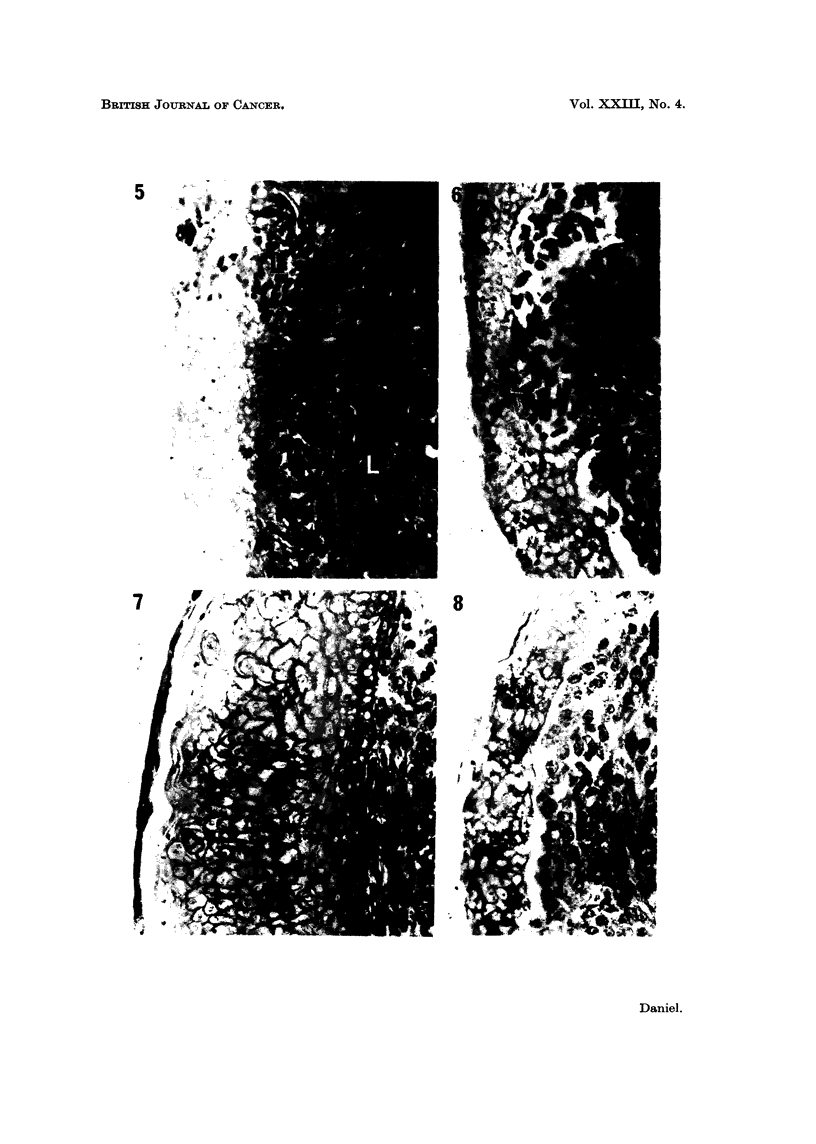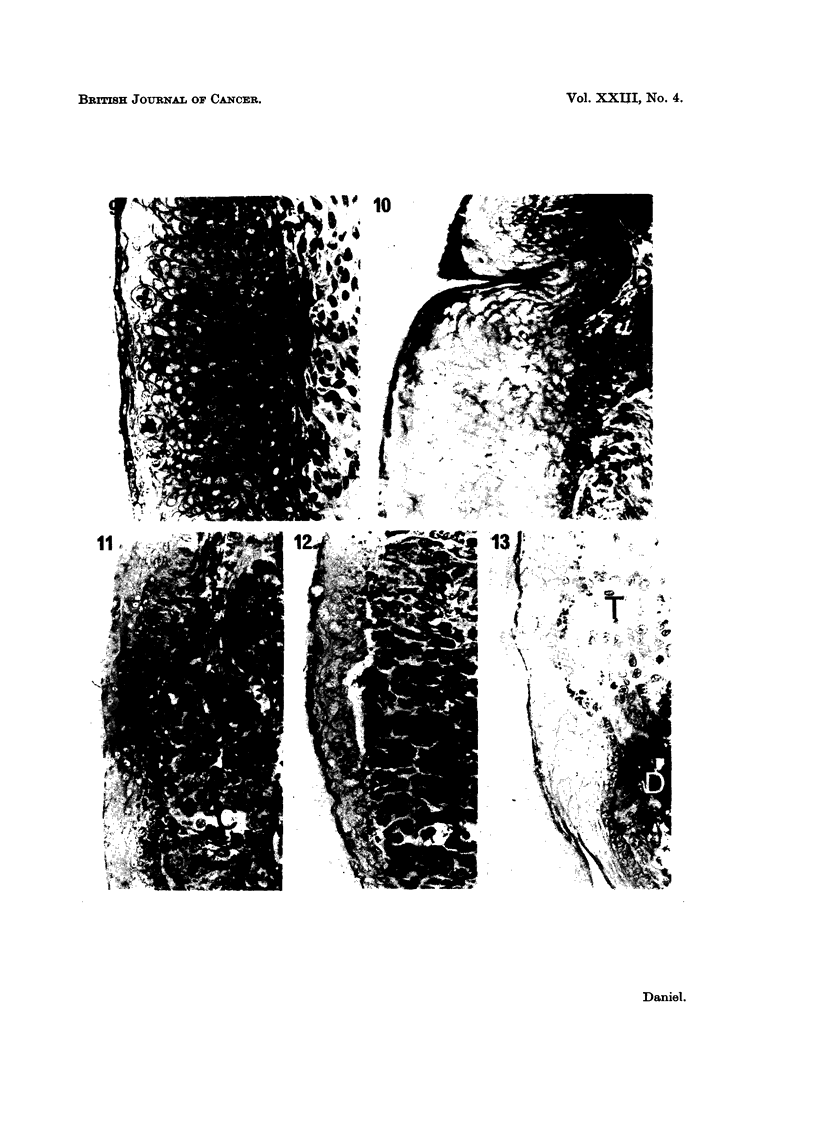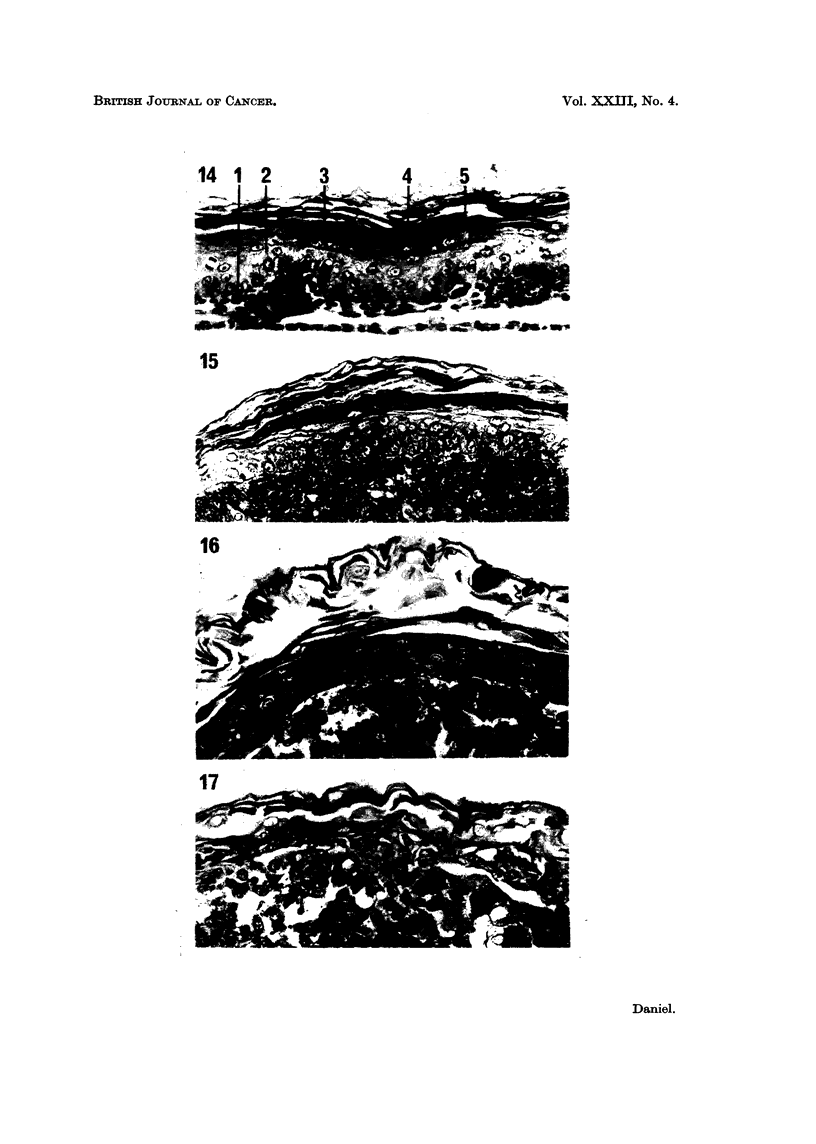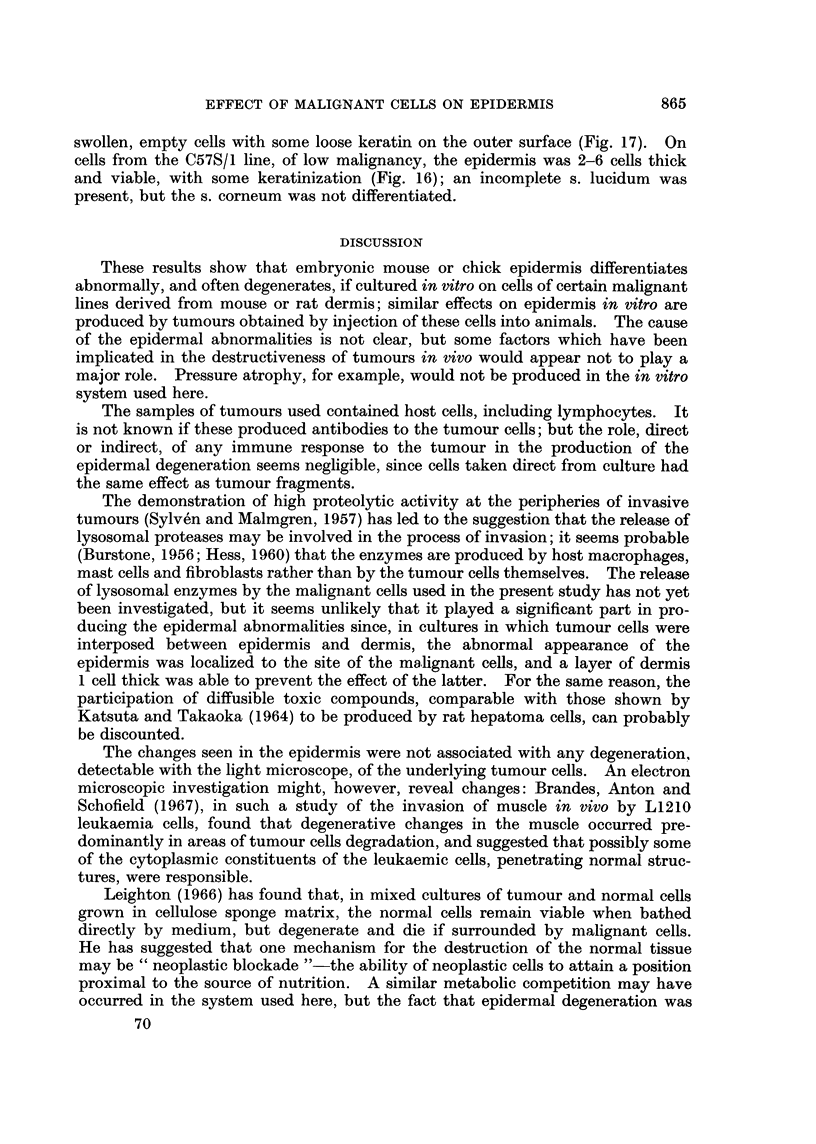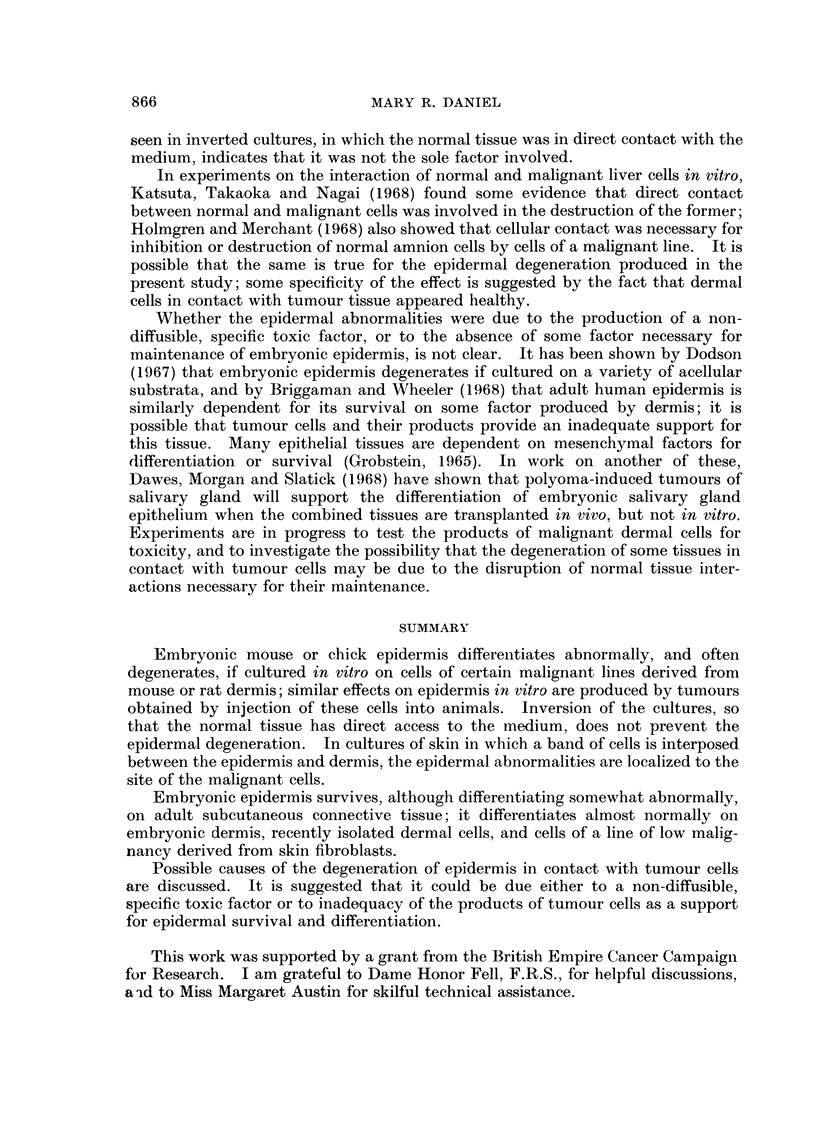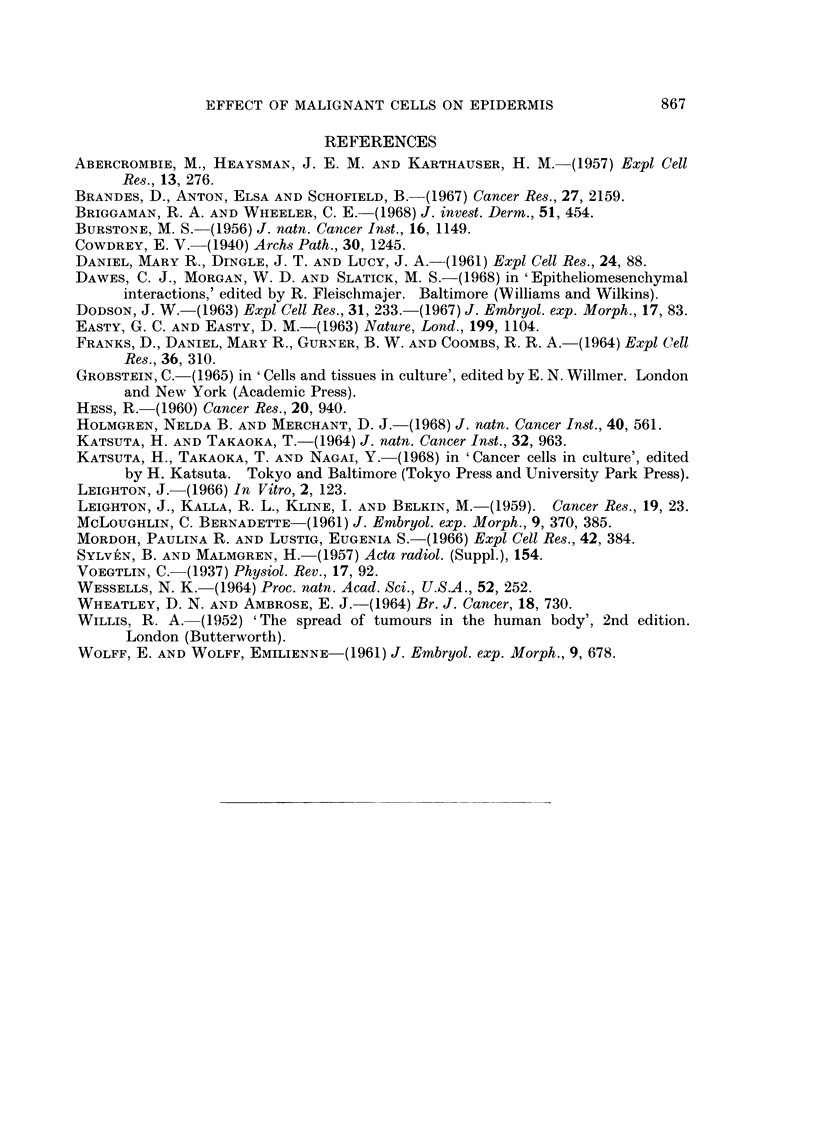# The effect of malignant dermal cells on embryonic epidermis in vitro.

**DOI:** 10.1038/bjc.1969.106

**Published:** 1969-12

**Authors:** M. R. Daniel

## Abstract

**Images:**


### THE EFFECT OF MALIGNANT DERMAL CELLS ON EMBRYONIC EPIDERMIS *IN VITRO*

MARY R. DANIEL

*From the Strangeways Research Laboratory, Cambridge*

Received for publication July 30, 1969

It is probable that several factors are involved in the destruction of normal tissue at the growing edge of a malignant tumour. Among the possible causes that have been suggested are pressure (Willis, 1952), the release of lytic enzymes (Sylvén and Malmgren, 1957), the uptake of cytoplasmic constituents released from degenerating tumour cells (Brandes, Anton and Schofield, 1967), side effects of an immune response by the host (Wheatley and Ambrose, 1964), toxic factors (Voegtlin, 1937; Katsuta and Takaoka, 1964), and competition for nutrients (Cowdry, 1940; Leighton, 1966).

The technique of organ culture has been used by some workers to study the problem of invasion. Following the demonstration by Abercrombie, Heaysman and Karthauser (1957) of the reduced contact inhibition of movement of tumour cells *in vitro*, and the work of Leighton, Kalla, Kline and Belkin (1959) on the mutual infiltration of normal fibroblasts and malignant cells, it has been shown by Easty and Easty (1963) that malignant cells penetrate between normal cells of various tissues in contiguous organ cultures. Wolff and Wolff (1961) have demonstrated that tumour cells cultured in contact with embryonic chick mesonephros destroy and replace the normal tissue.

In the present study, an investigation has been made of the effect of malignant fibroblasts, of dermal origin, on the differentiation of embryonic epidermis. This is easily obtained free of other tissue, and is known (McLoughlin, 1961; Dodson, 1963, 1967; Wessells, 1964) to depend for survival and differentiation on the presence of some factor(s) produced by normal dermis; Mordoh and Lustig (1966) have shown that the dermal effect is not species-specific. The effect on the epidermis of substituting malignant for normal dermal cells was therefore examined.

### MATERIALS AND METHODS

The fibroblastic cell lines used were:

C3HS/1, derived from the pooled dorsal skin of foetal C3H/He mice near term (Franks, Daniel, Gurner and Coombs, 1964) and grown in Waymouth's medium MB752/1 supplemented with 5% precolostral calf serum;

C3HS/1P, a subline of C3HS/1, adapted to grow in serum-free MB752/1 supplemented with 0·5% peptone (" Bactopeptone ", Difco);

C57S/1, derived from the pooled dorsal skin of foetal C57BL mice near term (Franks *et al.*, 1964) and grown in MB752/1 supplemented with 5% precolostral calf serum;

C57S/1P, a subline of C57S/1, adapted to grow in serum-free MB752/1 supplemented with 0·5% peptone;

16C, derived from the pooled dermal tissue of foetal Hooded rats near term (Daniel, Dingle and Lucy, 1961) and grown in MB752/1 supplemented with 5% precolostral calf serum.

Recently isolated fibroblasts, obtained by tryptic digestion of the pooled dorsal skin of mouse embryos near term, were used in some experiments; they were grown in MB752/1 supplemented with 15% precolostral calf serum.

On subcutaneous injection into animals of the corresponding strains, cells of the lines C3HS/1, C3HS/1P, C57S/1P and 16C produced tumours in 70 to 90% of the hosts within 1 month. Cells from the samples of line C57S/1 used produced tumours in only 30% of the hosts, after a latent period of from 3–4 months.

Fragments of the tumours produced by injection of the cells were used as substrata for the epidermis in some experiments; the effect of substituting adult subcutaneous tissue for embryonic dermis was also examined.

The epidermis used was from the anterior shank skin of 12-day chick embryos or the dorsal skin of 15- or 17-day foetal mice. It was separated from the dermis by treatment with a solution of 0·04% versene in phosphate-buffered salt solution, as described by Dodson (1963); the chick epidermis separated readily after 20 minutes' exposure to versene at room temperature, whereas the mouse skin required 1 hour's incubation at 37° C. For most of the experiments, the epidermis was spread on the tissue under investigation; when cells from culture were used, clumps of these were obtained either direct from monolayer culture of the line or from the more compact mass produced when the cells were grown for 1 or 2 days on millipore filter or agarose gel at the surface of the appropriate liquid medium. In some experiments, a ribbon of cells from a monolayer was laid on dermis from which the epidermis had been partially removed; the epidermis was then replaced, so that the cell band was interposed between epidermis and dermis within part of the specimen.

All cultures were grown on rayon gauze impregnated with 0·8% agarose gel (Seravac Laboratories Ltd., Maidenhead) and supported on stainless steel grids at the surface of the liquid medium. This was Waymouth's medium MB752/1, modified by the substitution of Hanks's balances salt solution for the original buffer system, and the reduction of the glucose content to 1 g./litre; it was supplemented with 0·5% peptone. The gas phase was air, with the $CO_2$ concentration raised to approximately 2%. The medium was renewed after 2 days, and the cultures were fixed, after 4 days, in Zenker's fluid. Paraffin sections 6 $\mu$ thick were stained with carmalum-aniline blue-orange G or by the periodic acid-Schiff technique (PAS).

### RESULTS

### *Chick epidermis*

As shown by Wessells (1964), the epidermis from 12-day chick embryos, when separated from and then recombined with its own dermis, showed, after 4 days' incubation in serum-free medium, a degree of differentiation comparable with that of control, untreated skin (Fig. 1). The columnar basal cells were separated from the dermis by a basement membrane which had a high affinity for aniline blue and was stained red by the PAS technique. The intermediate cell layer was 3–5 cells thick, with squamous outer cells. A periderm and secondary periderm were present, and a subperidermal layer was seen in most cultures.

Chick epidermis combined with dermis from the skin of foetal mice or rats near term, or with fibroblasts recently isolated from this tissue, differentiated normally, except that the basal cells were cubical rather than columnar, and a basement membrane was not always formed (Fig. 2). On adult mouse subcutaneous tissue, the epidermis did not produce a basement membrane. The cells of the basal layer were cubical or flattened, and some of the outer, squamous cells of the intermediate layer had thickened walls; the secondary periderm was usually cornified, and the subperidermal cells, when present, appeared empty (Fig. 3).

When the epidermis was incubated on pieces of the tumours produced in mice by injection of cells of the malignant line C3HS/1, no basement membrane was formed, and differentiation was grossly abnormal. In some specimens the cells of the basal layer were cubical or flattened, and often vacuolated; the intermediate layer was up to 20 cells thick (Fig. 4), and the outer layers consisted of swollen cells which did not stain with either of the techniques used. Secondary periderm and subperiderm were not always formed. In other cultures, the epidermis showed almost complete degeneration (Fig. 5) without any thickening; in some of these, tumour cells appeared to be infiltrating the dead tissue (Fig. 6).

Epidermis grown on C3HS/1 cells taken direct from culture showed essentially the same abnormalities as that on the corresponding tumour tissue. If the cells were from the line grown in medium containing calf serum, complete degeneration of the epidermis was rarely seen, and the cells of the intermediate layer, although large and irregular in outline, sometimes showed some cytoplasmic staining. The basal cells, however, were often vacuolated, no basement membrane was formed, and the tissue was usually thickened (Fig. 7). On cells from the subline C3HS/1P, which had been adapted to grow in serum-free medium, the epidermis sometimes degenerated completely within 4 days (Fig. 8), with little or no sign of an initial increase in the number of cell layers.

If the cultures were grown inverted, so that the peridermal surface of the epidermis was in contact with the nutrient medium, the basal cells were again vacuolated. In a few cultures (Fig. 9) the epidermis appeared viable, but was thickened, and the cells of the intermediate layer were large and irregular in shape, with thick walls. Most cultures, however, showed complete degeneration of the epidermis.

When a band or mass of C3HS/1 cells was interposed between epidermis and dermis, the epidermis overlying the malignant cells again showed abnormal differentiation and degeneration. Where the epidermis was in contact with normal dermis, on either side of the tumour cells, it differentiated normally (Fig. 10). The change in appearance of the epidermis with the change in substratum was often very sharp, although in some cultures normal development extended to the tissue overlying the tumour cells which were near to the dermis. The interposition of a single layer of dermal cells between the C3HS/1 cells and the epidermis resulted in normal differentiation of the latter; the dermal cells in contact with the tumour cells appeared healthy.

Epidermis grown on cells from line C57S/1, which produces tumours in C57BL mice only after a relatively long lag period of 3–4 months, remained viable, although a basement membrane was not formed and differentiation was not always complete. In some cultures, the cells of the basal layer were cubical or flattened, and no subperidermal layer was formed; in others (Fig. 11), the only difference from control cultures was that the basal cells were cubical rather than columnar. On the subline C57S/1P, grown in serum-free medium, and with a greatly increased malignancy, the epidermis degenerated completely within 4 days (Fig. 12). The same result was obtained when cells of the line of malignant rat dermal fibroblasts, 16C, were used as the substratum for the epidermis (Fig. 13).

*Mouse epidermis*

The epidermis from 15-day mouse embryos, when separated from and recombined with its own dermis, differentiated almost normally *in vitro*, although no hair follicles were formed. Samples fixed after 4 days showed a stratum germinativum, a stratum spinosum 2–3 cells thick, a stratum granulosum 3–4 cells thick, a stratum lucidum, a thick stratum corneum and a layer of loose keratin (Fig. 14). The epidermis from 17-day foetuses, after 4 days' *in vitro* in combination with dermis, was thinner, and contained no distinguishable s. granulosum. Epidermis grown on recently isolated dermal fibroblasts resembled tissue of the same age grown on intact dermis (Fig. 15).

Epidermis cultured on cells from the malignant lines C3HS/1, C3HS/1P, C57S/1P or 16C, or on pieces of the tumours produced by these, degenerated almost completely within 4 days. In some cultures a few viable though flattened basal cells were present, but in most the epidermis consisted of a few layers of

---

EXPLANATION OF PLATES

All stained with carmalum-aniline blue-orange G. ×300.

FIG. 1–13.—Embryonic chick epidermis.

FIG. 1.—On embryonic chick dermis. BM—basement membrane; B—basal cells; I—intermediate cells; SP—subperiderm; 2P—secondary periderm.
FIG. 2.—On foetal mouse dermis. Basal cells cubical, differentiation otherwise normal.
FIG. 3.—On adult mouse adipose tissue. Basal cells cubical or flattened. Subperidermal cells empty. Periderm cornified.
FIG. 4.—On C3HS/1 tumour. Basal cells and adjacent intermediate cells flattened. Intermediate layer 26 cells thick, cells swollen and empty.
FIG. 5.—On C3HS/1 tumour. Host lymphocytes present (L). Vacuolation of basal cells and some intermediate cells; other intermediate cells swollen and empty.
FIG. 6.—On C3HS/1 tumour. Complete degeneration of epidermis. Possible infiltration by tumour cells (arrow).
FIG. 7.—On C3HS/1 cells. Basal and adjacent intermediate cells vacuolated. Outer intermediate cells swollen and empty. Secondary periderm cornified.
FIG. 8.—On C3HS/1P cells. Complete degeneration of epidermis.
FIG. 9.—Under C3HS/1 cells. Vacuolation of basal cells. Intermediate layer thickened; cells swollen, with thick walls. Secondary periderm present.
FIG. 10.—On C3HS/1 cells (T) and dermis (D). Differentiation essentially normal on dermis, abnormal on adjacent tumour cells.
FIG. 11.—On C57S/1 cells (C) and dermis (D). Differentiation essentially normal throughout.
FIG. 12.—On C57S/1P cells. Complete degeneration of epidermis.
FIG. 13.—On 16C cells (T) and dermis (D). Sharp demarcation between degenerated epidermis overlying tumour cells, and normal epidermis overlying dermis.

FIG. 14–17.—Foetal mouse epidermis.

FIG. 14.—On foetal mouse dermis. 1—s. germinativum; 2—s. spinosum; 3—s. granulosum, 4—s. lucidum; 5—s. corneum.
FIG. 15.—On recently isolated mouse dermal cells. Essentially normal differentiation.
FIG. 16.—On C57S/1. No s. corneum present, but tissue otherwise normal.
FIG. 17.—On C57S/1P. Complete degeneration, with some keratinization.

Daniel.

Daniel.

Daniel.

Daniel.

swollen, empty cells with some loose keratin on the outer surface (Fig. 17). On cells from the C57S/1 line, of low malignancy, the epidermis was 2–6 cells thick and viable, with some keratinization (Fig. 16); an incomplete s. lucidum was present, but the s. corneum was not differentiated.

### DISCUSSION

These results show that embryonic mouse or chick epidermis differentiates abnormally, and often degenerates, if cultured *in vitro* on cells of certain malignant lines derived from mouse or rat dermis; similar effects on epidermis *in vitro* are produced by tumours obtained by injection of these cells into animals. The cause of the epidermal abnormalities is not clear, but some factors which have been implicated in the destructiveness of tumours *in vivo* would appear not to play a major role. Pressure atrophy, for example, would not be produced in the *in vitro* system used here.

The samples of tumours used contained host cells, including lymphocytes. It is not known if these produced antibodies to the tumour cells; but the role, direct or indirect, of any immune response to the tumour in the production of the epidermal degeneration seems negligible, since cells taken direct from culture had the same effect as tumour fragments.

The demonstration of high proteolytic activity at the peripheries of invasive tumours (Sylvén and Malmgren, 1957) has led to the suggestion that the release of lysosomal proteases may be involved in the process of invasion; it seems probable (Burstone, 1956; Hess, 1960) that the enzymes are produced by host macrophages, mast cells and fibroblasts rather than by the tumour cells themselves. The release of lysosomal enzymes by the malignant cells used in the present study has not yet been investigated, but it seems unlikely that it played a significant part in producing the epidermal abnormalities since, in cultures in which tumour cells were interposed between epidermis and dermis, the abnormal appearance of the epidermis was localized to the site of the malignant cells, and a layer of dermis 1 cell thick was able to prevent the effect of the latter. For the same reason, the participation of diffusible toxic compounds, comparable with those shown by Katsuta and Takaoka (1964) to be produced by rat hepatoma cells, can probably be discounted.

The changes seen in the epidermis were not associated with any degeneration, detectable with the light microscope, of the underlying tumour cells. An electron microscopic investigation might, however, reveal changes: Brandes, Anton and Schofield (1967), in such a study of the invasion of muscle *in vivo* by L1210 leukaemia cells, found that degenerative changes in the muscle occurred predominantly in areas of tumour cells degradation, and suggested that possibly some of the cytoplasmic constituents of the leukaemic cells, penetrating normal structures, were responsible.

Leighton (1966) has found that, in mixed cultures of tumour and normal cells grown in cellulose sponge matrix, the normal cells remain viable when bathed directly by medium, but degenerate and die if surrounded by malignant cells. He has suggested that one mechanism for the destruction of the normal tissue may be " neoplastic blockade "—the ability of neoplastic cells to attain a position proximal to the source of nutrition. A similar metabolic competition may have occurred in the system used here, but the fact that epidermal degeneration was seen in inverted cultures, in which the normal tissue was in direct contact with the medium, indicates that it was not the sole factor involved.

In experiments on the interaction of normal and malignant liver cells *in vitro*, Katsuta, Takaoka and Nagai (1968) found some evidence that direct contact between normal and malignant cells was involved in the destruction of the former; Holmgren and Merchant (1968) also showed that cellular contact was necessary for inhibition or destruction of normal amnion cells by cells of a malignant line. It is possible that the same is true for the epidermal degeneration produced in the present study; some specificity of the effect is suggested by the fact that dermal cells in contact with tumour tissue appeared healthy.

Whether the epidermal abnormalities were due to the production of a non-diffusible, specific toxic factor, or to the absence of some factor necessary for maintenance of embryonic epidermis, is not clear. It has been shown by Dodson (1967) that embryonic epidermis degenerates if cultured on a variety of acellular substrata, and by Briggaman and Wheeler (1968) that adult human epidermis is similarly dependent for its survival on some factor produced by dermis; it is possible that tumour cells and their products provide an inadequate support for this tissue. Many epithelial tissues are dependent on mesenchymal factors for differentiation or survival (Grobstein, 1965). In work on another of these, Dawes, Morgan and Slatick (1968) have shown that polyoma-induced tumours of salivary gland will support the differentiation of embryonic salivary gland epithelium when the combined tissues are transplanted *in vivo*, but not *in vitro*. Experiments are in progress to test the products of malignant dermal cells for toxicity, and to investigate the possibility that the degeneration of some tissues in contact with tumour cells may be due to the disruption of normal tissue interactions necessary for their maintenance.

### SUMMARY

Embryonic mouse or chick epidermis differentiates abnormally, and often degenerates, if cultured *in vitro* on cells of certain malignant lines derived from mouse or rat dermis; similar effects on epidermis *in vitro* are produced by tumours obtained by injection of these cells into animals. Inversion of the cultures, so that the normal tissue has direct access to the medium, does not prevent the epidermal degeneration. In cultures of skin in which a band of cells is interposed between the epidermis and dermis, the epidermal abnormalities are localized to the site of the malignant cells.

Embryonic epidermis survives, although differentiating somewhat abnormally, on adult subcutaneous connective tissue; it differentiates almost normally on embryonic dermis, recently isolated dermal cells, and cells of a line of low malignancy derived from skin fibroblasts.

Possible causes of the degeneration of epidermis in contact with tumour cells are discussed. It is suggested that it could be due either to a non-diffusible, specific toxic factor or to inadequacy of the products of tumour cells as a support for epidermal survival and differentiation.

This work was supported by a grant from the British Empire Cancer Campaign for Research. I am grateful to Dame Honor Fell, F.R.S., for helpful discussions, and to Miss Margaret Austin for skilful technical assistance.

### REFERENCES

Abercrombie, M., Heaysman, J. E. M. and Karthauser, H. M.—(1957) *Expl Cell Res.*, **13**, 276.
Brandes, D., Anton, Elsa and Schofield, B.—(1967) *Cancer Res.*, **27**, 2159.
Briggaman, R. A. and Wheeler, C. E.—(1968) *J. invest. Derm.*, **51**, 454.
Burstone, M. S.—(1956) *J. natn. Cancer Inst.*, **16**, 1149.
Cowdrey, E. V.—(1940) *Archs Path.*, **30**, 1245.
Daniel, Mary R., Dingle, J. T. and Lucy, J. A.—(1961) *Expl Cell Res.*, **24**, 88.
Dawes, C. J., Morgan, W. D. and Slatick, M. S.—(1968) in 'Epitheliomesenchymal interactions,' edited by R. Fleischmajer. Baltimore (Williams and Wilkins).
Dodson, J. W.—(1963) *Expl Cell Res.*, **31**, 233.—(1967) *J. Embryol. exp. Morph.*, **17**, 83.
Easty, G. C. and Easty, D. M.—(1963) *Nature, Lond.*, **199**, 1104.
Franks, D., Daniel, Mary R., Gurner, B. W. and Coombs, R. R. A.—(1964) *Expl Cell Res.*, **36**, 310.
Grobstein, C.—(1965) in 'Cells and tissues in culture', edited by E. N. Willmer. London and New York (Academic Press).
Hess, R.—(1960) *Cancer Res.*, **20**, 940.
Holmgren, Nelda B. and Merchant, D. J.—(1968) *J. natn. Cancer Inst.*, **40**, 561.
Katsuta, H. and Takaoka, T.—(1964) *J. natn. Cancer Inst.*, **32**, 963.
Katsuta, H., Takaoka, T. and Nagai, Y.—(1968) in 'Cancer cells in culture', edited by H. Katsuta. Tokyo and Baltimore (Tokyo Press and University Park Press).
Leighton, J.—(1966) *In Vitro*, **2**, 123.
Leighton, J., Kalla, R. L., Kline, I. and Belkin, M.—(1959). *Cancer Res.*, **19**, 23.
McLoughlin, C. Bernadette—(1961) *J. Embryol. exp. Morph.*, **9**, 370, 385.
Mordoh, Paulina R. and Lustig, Eugenia S.—(1966) *Expl Cell Res.*, **42**, 384.
Sylvén, B. and Malmgren, H.—(1957) *Acta radiol.* (Suppl.), **154**.
Voegtlin, C.—(1937) *Physiol. Rev.*, **17**, 92.
Wessells, N. K.—(1964) *Proc. natn. Acad. Sci., U.S.A.*, **52**, 252.
Wheatley, D. N. and Ambrose, E. J.—(1964) *Br. J. Cancer*, **18**, 730.
Willis, R. A.—(1952) 'The spread of tumours in the human body', 2nd edition. London (Butterworth).
Wolff, E. and Wolff, Emilienne—(1961) *J. Embryol. exp. Morph.*, **9**, 678.